# Non-anatomical reconstruction of lateral ulnar collateral ligament of the elbow after tumor resection

**DOI:** 10.1007/s11751-015-0235-1

**Published:** 2015-11-17

**Authors:** Masuo Hanada, H. Kadota, T. Matsunobu, E. Shimada, Y. Iwamoto

**Affiliations:** Department of Plastic Surgery, Kyushu University, 3-1-1, Maidashi, Higashi-ku, Fukuoka, 812-8582 Japan; Department of Orthopedic Surgery, Kyushu University, 3-1-1, Maidashi, Higashi-ku, Fukuoka, 812-8582 Japan

**Keywords:** Elbow, Lateral ulnar collateral ligament reconstruction, Radial forearm flap, Tumor wide resection

## Abstract

We present the case of an 80-year-old man with a tumor recurrence on his right arm 6 years after initial treatment. The lateral aspect of the elbow joint, involving overlaying skin, muscles, tendons, joint capsule, lateral collateral ligament complex, the lateral 1/3 of the capitellum, and lateral epicondyle of humerus were excised in the tumor resection. Intraoperative assessment revealed multidirectional instability of the elbow, and joint stabilization was needed. Because the lateral epicondyle was resected, graft placement in an anatomical position was impossible to carry out. Therefore, non-anatomical reconstruction of lateral ulnar collateral ligament with palmaris longus tendon graft was performed. The skin was reconstructed using an antegrade pedicled radial forearm flap. For wrist extension reconstruction, the pronator quadratus tendon was transferred to the extensor carpi radialis brevis tendon. One year after the operation, elbow range of motion was 5–130°. The patient remains symptom free. The Mayo elbow performance score is good. The Musculoskeletal Tumor Society rating score is excellent. To our knowledge, this is the first report of an elbow lateral ulnar collateral ligament reconstruction after tumor resection.

## Introduction

Amputation used to be the primary treatment for patients with soft tissue tumors of the extremities [1]. With the application of multimodal treatment strategies, most patients with localized extremity soft tissue tumors now undergo limb-sparing treatment [2]. Using reconstructive techniques such as prosthesis and flap surgery, we are able to even spare the limbs of patients who have recurrent bone and soft tissue tumors [3].

Because the elbow is an uncommon site for primary or metastatic bone tumors, there is little information about elbow reconstruction after tumor resection [4]. Reconstruction techniques include autografts, allografts, and endoprosthesis [3]. Elbow allograft is not recommended for routine use because of a high complication rate [5]. Endoprosthetic reconstruction has a high infection rate because of the immunocompromised nature of sarcoma patients [6]. Autograft is the ideal reconstructive procedure, but has significant limitations, including donor site morbidity, inadequate amount of graft material, and inappropriate form. Careful consideration of appropriate surgical procedures for elbow reconstruction is needed.

To our knowledge, there are no reports of elbow lateral ulnar collateral ligament (LUCL) reconstruction after tumor resection.

## Case report

An 80-year-old man visited our hospital complaining of a painless mass in his right arm 6 years after initial tumor resection with wide margins. There were two palpable masses near the elbow joint. MRI revealed T1 iso T2 high masses in the posterior portion of his right arm. Pathological examination of the needle biopsy specimen revealed the recurrence of the original tumor (extraskeletal osteosarcoma).

The patient exhibited depression and mild cognitive impairment. He had difficulty in daily activities, and lived in a nursing home. Amputation may have improved the local disease control, but would not improve survival under these circumstances [2]. He and his family chose limb-sparing surgery. Wide resection of the recurrent tumor and reconstruction was planned.

**Fig. 1:**
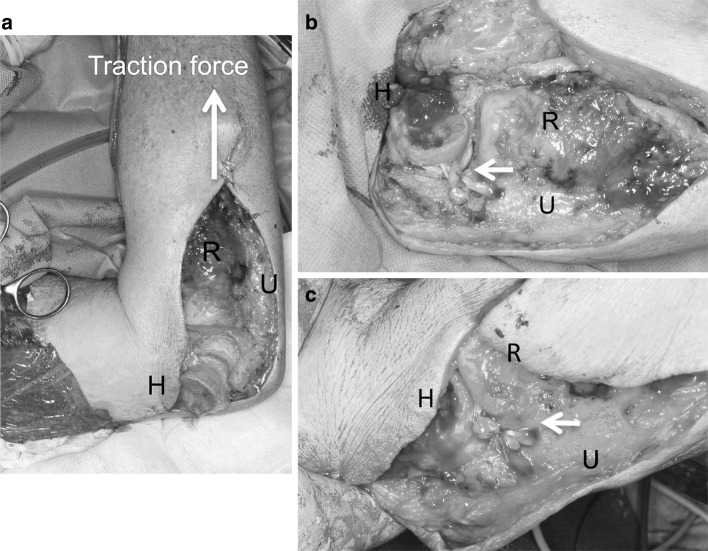
Intraoperative view of the elbow joint. **a** Before ligament reconstruction, **b** after ligament reconstruction in elbow flexion, **c** in extension. **a** Ulnohumeral joint was opened widely and the ulna rotated around the humerus. There was no tissue above the lateral part of the elbow joint. **b**, **c** Two drill holes for the graft insertion were made in posterior articular surface of capitellum. The ulnar bone holes were placed near the insertion of annular ligament. *White arrow* shows reconstructed LUCL: *H* humerus, *R* radial head, *U* ulnar head

An orthopedic oncologist performed a wide resection. To accomplish this, overlaying skin, brachioradialis, supinator extensor muscles, lateral half of the triceps tendon, joint capsule, lateral collateral ligament complex, the lateral 1/3 of the capitellum, and lateral epicondyle of humerus were resected. As a result of tumor wide resection, there was no tissue above the lateral part of the elbow joint, and the elbow suffered gross varus instability (Fig. 1a). The ulnohumeral joint opened widely when traction was applied to the forearm with the elbow joint in 90° flexion (Fig. 1a). Under rotational force, the ulna easily rotated medially and laterally around the humerus (Fig. 1a).

Subsequently, the LUCL was reconstructed with a palmaris longus tendon graft. Because the lateral epicondyle was resected, graft placement in an anatomical position was impossible to carry out. Therefore, non-anatomical reconstruction of LUCL was performed. Two drill holes for the graft insertion were made in posterior articular surface of capitellum. To prevent impaction of the grafted tendon against the radial head during elbow extension, the ulnar bone tunnel was placed near the insertion of annular ligament (Fig. 1b, c). Full range of motion of the elbow joint and tightness of the planned tendon graft were confirmed with a suture placed through the bone tunnel. The graft was passed through the bone tunnel in a figure-of-eight configuration and sutured together with 3-0 FiberWire (Arthrex, Naples, FL, USA) (Fig. 1b, c).

The pronator quadratus tendon was transferred to the extensor carpi radialis brevis (ECRB) tendon for wrist extension reconstruction. An antegrade pedicled radial forearm flap was raised and covered the exposed joint. The flap donor site was repaired with a full-thickness skin graft.

The postoperative course was uneventful. The flap completely survived. Initially, the elbow was placed in 90° of flexion with the forearm in a neutral position. Six weeks after surgery, the patient was allowed protected movement of the elbow under the guidance of an experienced hand therapist. The patient was advised not to push up or hold heavy objects (over 2 kg) with the affected hand for 6 months. Tumor recurrence occurred 11 months postoperatively, and a marginal resection was performed.

**Fig. 2:**
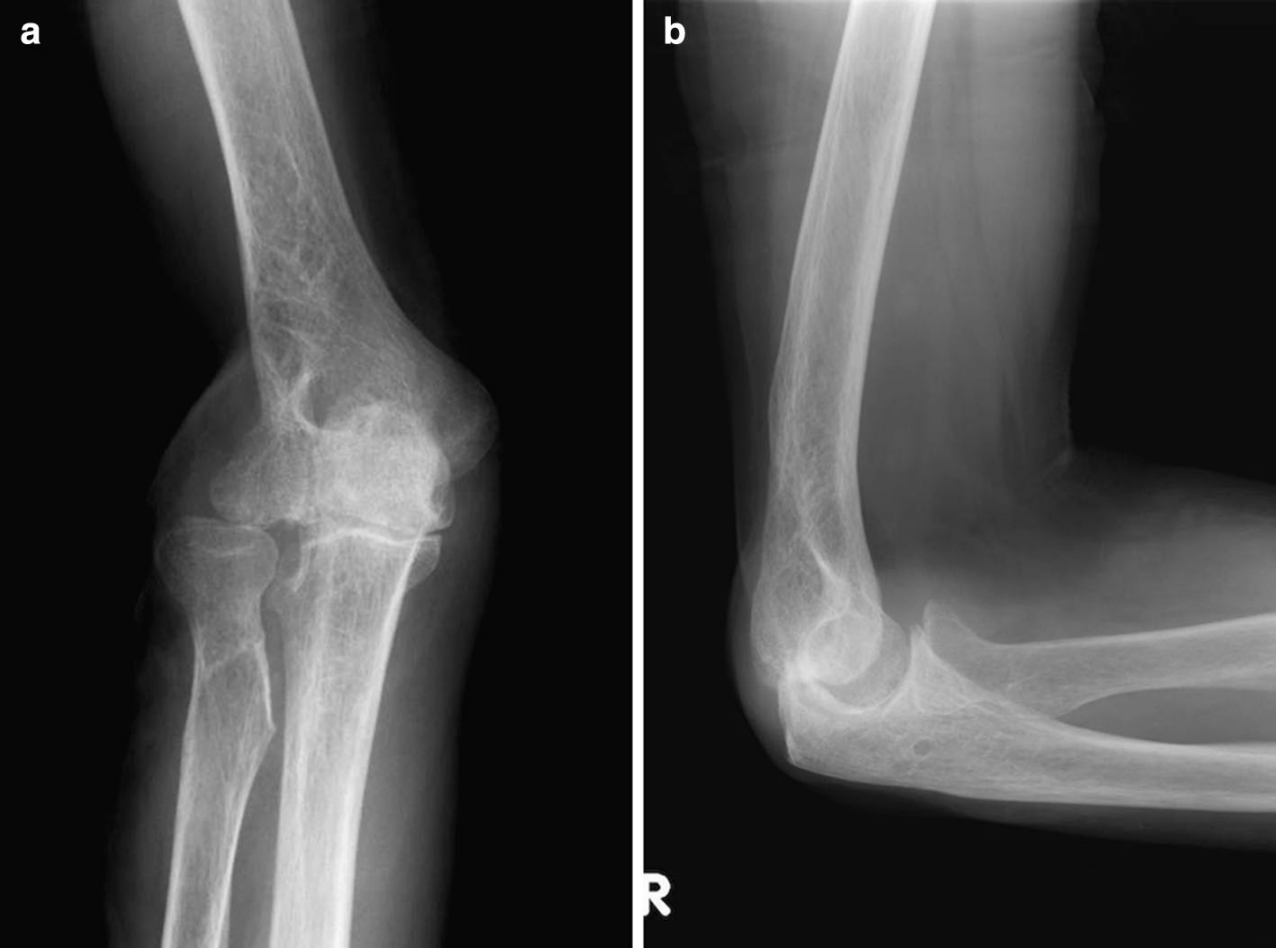
X-rays. **a** Anteroposterior and **b** lateral. Degenerative changes in the elbow joint, especially in the radiohumeral joint, were observed 1 year after surgery

**Fig. 3:**
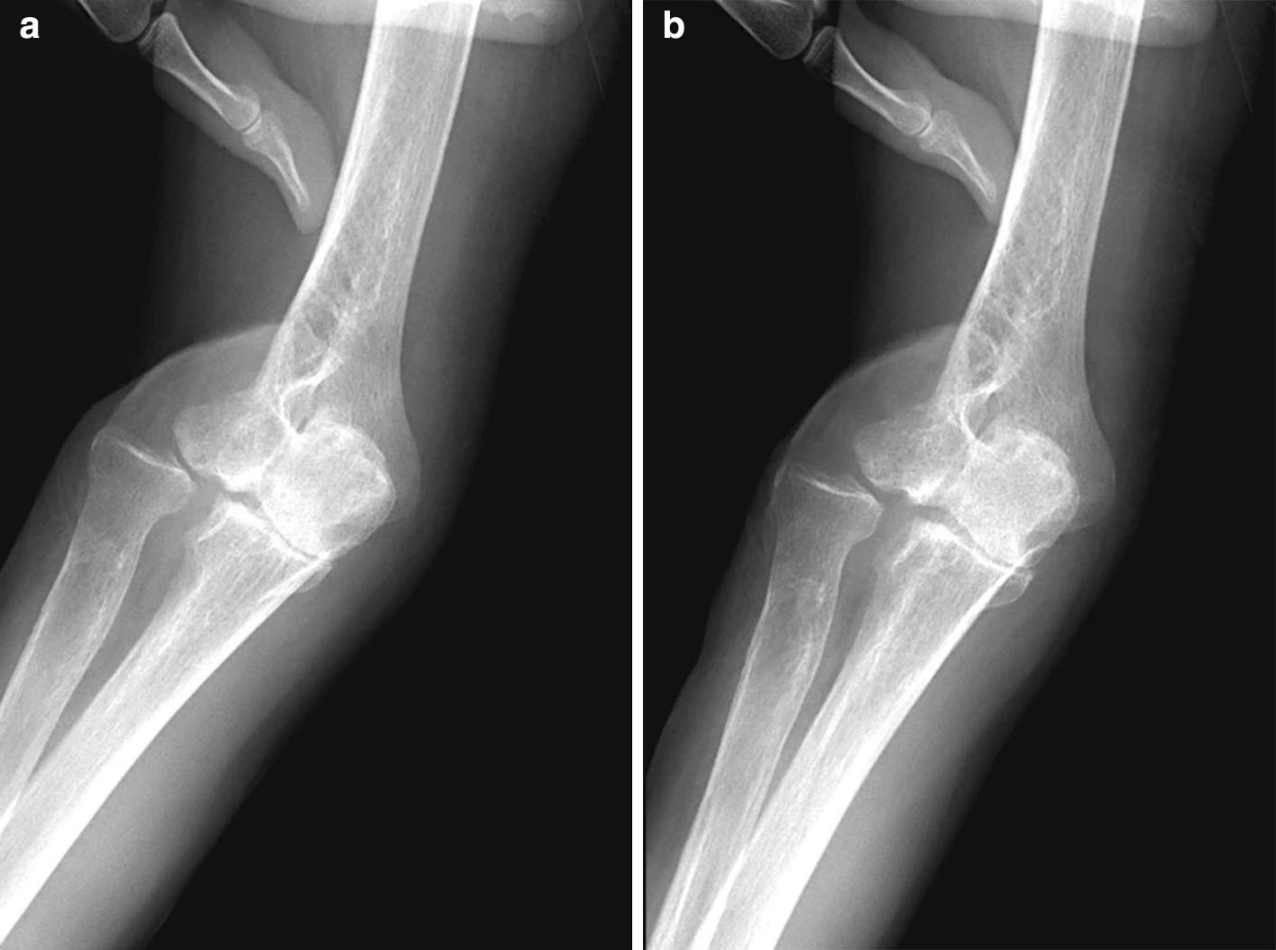
Stress examination under fluoroscopy. **a** Valgus and **b** varus stress. The elbow joint showed 10° valgus-varus instability

**Fig. 4:**
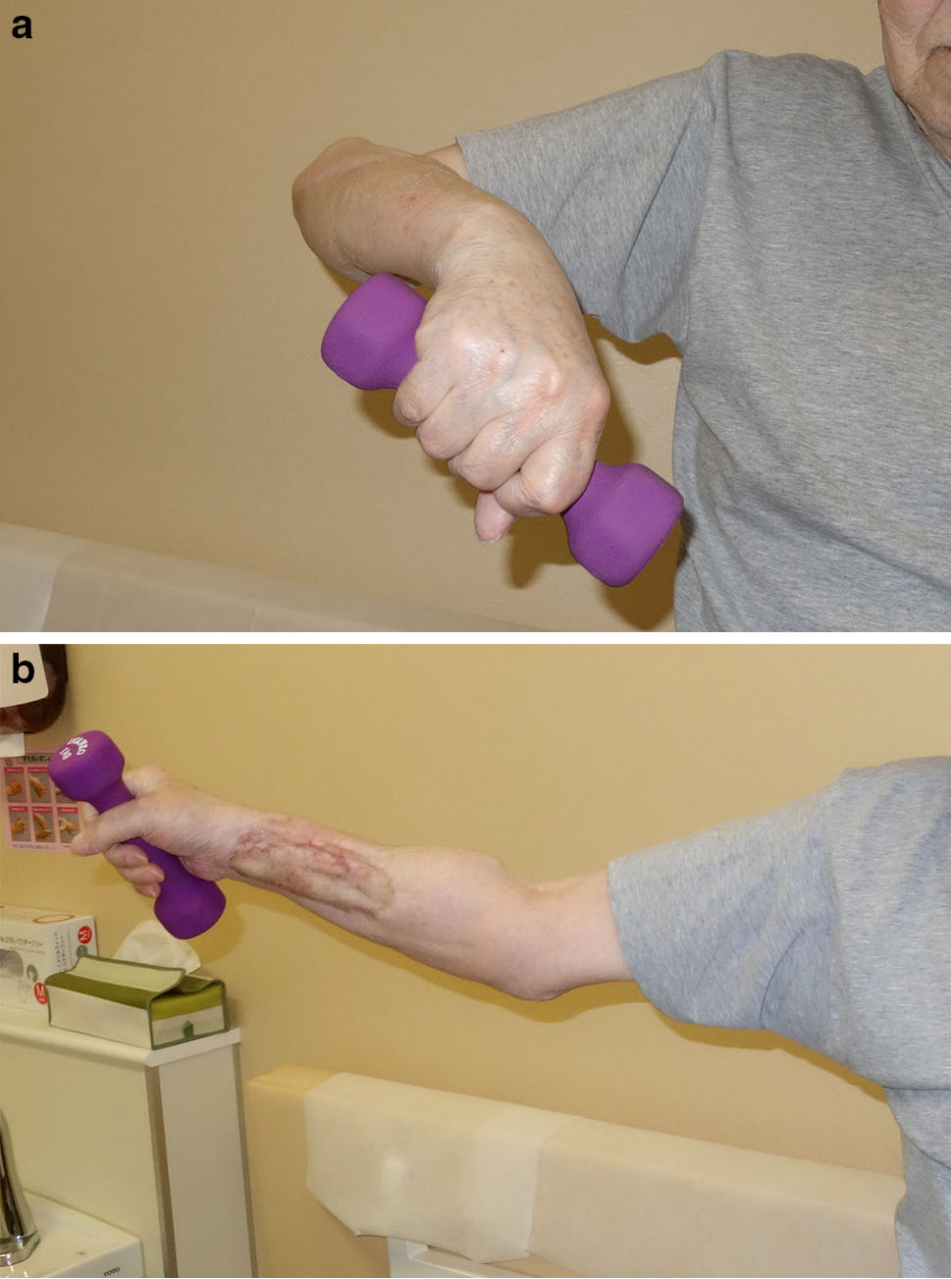
Clinical pictures. With the shoulder in abduction, the patient can hold a 1-kg object, and **a** flex and **b** extend his elbow joint

One year after surgery, separation of radius and ulna at the proximal radioulnar joint (PRUJ) was apparent, and degenerative change in the elbow joint was observed, especially in the radiohumeral joint (Fig. 2). Stress examination revealed 10° valgus-varus instability (Fig. 3). Elbow ROM was 5–130°. The active ROM at the wrist was 30° extension, 20° flexion, 45° supination, and 90° pronation. The Mayo elbow performance score was 85 (good). The Musculoskeletal Tumor Society rating score was excellent (26/30). With the shoulder in abduction, the patient can hold 1-kg objects, and flex and extend his elbow joint against tensile varus force on the lateral aspect of the elbow (Fig. 4). He claims to have no symptoms around the elbow joint and has no discomfort doing daily activities.

## Discussion

We performed three reconstructive procedures: joint coverage, wrist extensor reconstruction, and ligament reconstruction. For elbow joint coverage, the pedicled radial forearm flap is a reliable method without using a microsurgical procedure [7]. For wrist extension reconstruction, pronator quadratus transfer to the ECRB tendon is established treatment [8].

Intraoperative assessment revealed multidirectional instability of the elbow joint. Absence of the lateral epicondyle made it impossible to place the graft in an anatomical position. Therefore, we performed non-anatomical reconstruction in reference to the operative method for ligamentous reconstruction for posterolateral rotary instability of the elbow [9]. Without reconstruction, gross instability would remain, and the patient would not be able to hold his forearm against the gravity with the shoulder abducted. With ligament reconstruction, he can hold an object when the shoulder is abducted against tensile varus force on the lateral aspect of the elbow. The functional score is excellent or good. He has no discomfort.

One-year postoperative X-ray showed 10° valgus-varus instability, the separation of radius and ulna at the PRUJ, and degenerative change in the elbow joint. These observations show that the joint is not stabilized completely with the non-anatomical ligament reconstruction performed in this case. Careful follow-up examination is needed.

Another possible procedure to stabilize the joint is endoprosthetic reconstruction. While endoprosthesis achieves excellent stability, we consider that the risk of prosthesis joint infection is very high in this patient for the following reasons: (1) his old age and cognitive impairment, (2) extensive soft tissue dissection during tumor resection, and (3) long operating time for flap and tendon transfer. In addition, partial necrosis of radial forearm flap occasionally occurs [7]. Even small area of necrosis can cause implant exposure and subsequent infection. Endoprosthetic infection leads to repetitive debridement and lengthy hospital stay. If it had occurred, considering this patient’s old age and mental status, it would have been difficult to spare his right forearm.

In conclusion, this case report suggests that non-anatomical LUCL reconstruction after tumor wide resection makes the elbow joint painless and functional. The biological reconstruction performed in this patient avoided the possible complications related to endoprosthesis. However, the joint was not stabilized completely. We recommend that this procedure be performed in older patients or patients with complications to maintain their quality of life.
